# Association between smoking and glycemic control in men with newly diagnosed type 2 diabetes: a retrospective matched cohort study

**DOI:** 10.1080/07853890.2022.2075559

**Published:** 2022-05-16

**Authors:** Hon-Ke Sia, Chew-Teng Kor, Shih-Te Tu, Pei-Yung Liao, Jiun-Yi Wang

**Affiliations:** a Division of Endocrinology and Metabolism, Department of Internal Medicine, Changhua Christian Hospital, Changhua, Taiwan; b Department of Healthcare Administration, Asia University, Wufeng, Taiwan; c Department of Post-Baccalaureate Medicine, College of Medicine, National Chung Hsing University, Taichung, Taiwan; d School of Medicine, Kaohsiung Medical University, Kaohsiung, Taiwan; e Internal Medicine Research Center, Changhua Christian Hospital, Changhua, Taiwan; f Department of Medical Research, China Medical University Hospital, China Medical University, Taichung, Taiwan

**Keywords:** Smoking, glycemic control, newly diagnosed, diabetes, BMI

## Abstract

**Background:**

Longitudinal data on the association between smoking and glycemic control in men with newly diagnosed type 2 diabetes (T2DM) is scarce. Therefore, this study aimed to examine the extent of the association between smoking and glycemic control in this population.

**Methods:**

The retrospective cohort study identified 3044 eligible men with T2DM in a medical centre in Taiwan between 2002 and 2017. Smokers (*n* = 757) were matched 1:1 with non-smokers using propensity score-matching. All of them were followed for one year. Glycated haemoglobin (HbA1c) levels were measured at 0, 3, 6, 9, and 12 months after enrolment. Generalised estimating equations were used to assess smoking status-by-time interaction to determine the difference in HbA1c reduction between the two cohorts. All analyses were performed in 2020.

**Results:**

The estimated maximal difference in HbA1c reduction between smokers and non-smokers was 0.33% (95% CI, 0.05–0.62%) at 3 months of follow-up. For patients with body mass index (BMI) <25 kg/m^2^, the difference in HbA1c reduction between smokers and non-smokers was much larger (0.74%, 95% CI, 0.35–1.14%) than in those with a higher BMI.

**Conclusions:**

Our findings show that smoking was independently associated with unfavourable glycemic control among men with newly diagnosed T2DM, and such a detrimental association could be stronger in men with a lower BMI.

## Introduction

Smoking as an unhealthy behaviour is a global health issue, and according to estimates from the World Health Organisation (WHO) in 2018, 33% of men and 5% of women in the world smoke [1]. In Taiwan, comprehensive multisectoral national strategies and tobacco control action plans have strengthened legislation aimed at tobacco hazard prevention and the smoking rate decreased from 42.9% to 23.4% in men and from 4.6% to 2.4% in women from 2004 to 2018 [2]. Despite this decline, smoking continues to be a major health threat to men.

Diabetes is a widespread chronic disease with increasing prevalence worldwide, and poor glycemic control leads to micro- and macrovascular complications [3]. Smoking has been shown to increase not only the risk of vascular complications in individuals with type 2 diabetes (T2DM) but also diabetes incidence in the general population [4–6]. Smoking may directly affect glucose homeostasis through several mechanisms, such as increasing insulin resistance, decreasing insulin secretion, or impairing pancreatic beta cell function [7,8]. Previous studies have shown an adverse association between smoking and glycemic control in people with T2DM [9–12]. Of note, however, these studies employed a cross-sectional design that could not help determine the causal effect, and they did not focus on people newly diagnosed with diabetes.

Given that achieving better glycemic control in the first year of newly diagnosed diabetes can reduce the long-term risk of complications by the so-called “legacy effect,” these people are at a critical juncture for changing their health-related behaviours just after their diagnosis [3]. Therefore, more evidence from real-world data is warranted to clarify the relationship between smoking and glycemic control to improve the care of these people. As smoking is more prevalent in men than women, this study aimed to longitudinally examine the extent of association between smoking and glycemic control in men newly diagnosed with T2DM.

## Methods

### Data sources

This retrospective cohort study used data from the electronic medical record system of Changhua Christian Hospital (CCH), Taiwan, including laboratory data, prescriptions, the CCH research database, and the Diabetes Care Management Program (DCMP) diabetes registry. The DCMP, conducted at the Diabetes Care Centre of CCH, provides standardised comprehensive diabetes care including lifestyle assessment, physical examination, laboratory testing, and standardised one-on-one diabetes self-management (DSM) education by a coordinated multidisciplinary team. Diabetes specialists referred people with diabetes to the Diabetes Care Centre to participate in the DCMP, usually 2–6 weeks after the first outpatient clinic visit. A detailed description of the program has been reported elsewhere [13].

The study method conformed to relevant guidelines and regulations. The Institutional Review Board of CCH approved the study (IRB No: 191212). The requirement of informed consent was waived because the secondary data set used in the present study is anonymous and retrospectively retrieved.

### Study participants

Study participants were people with newly diagnosed T2DM. The range of time from the diagnosis of diabetes to the enrolment in DCMP was within one year. A total of 24473 people with T2DM participating in the DCMP during the period between January 2002 and December 2017 were screened for eligibility. Diagnosis of diabetes was based on the criteria established by the American Diabetes Association [14], including a fasting plasma glucose value ≥126 mg/dL, a 2-hour plasma glucose ≥200 mg/dL during a 75-g oral glucose tolerance test, random plasma glucose ≥200 mg/dL in people with classic symptoms of hyperglycaemia, or a glycated haemoglobin (HbA1c) level of ≥6.5%.

**Figure 1.**
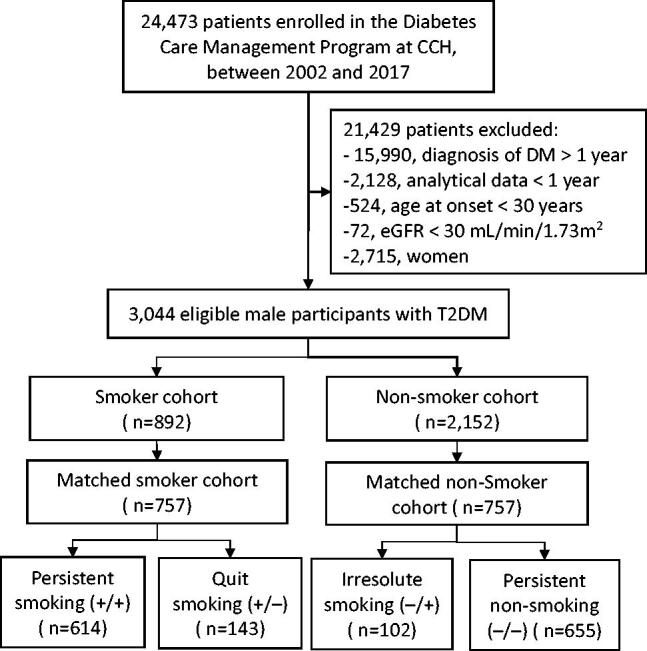
Flowchart of the study population. Eligible participants were matched based on the propensity score procedure. Abbreviations: CCH, Changhua Christian Hospital; T2DM, type 2 diabetes mellitus; eGFR, estimated glomerular filtration rate.

Individuals who were women (*n* = 2715), with <1 year of analysable data (*n* = 2128), whose diabetes duration was longer than 12 months (*n* = 15,990), and those younger than 30 years of age (with a greater likelihood of type 1 rather than type 2 diabetes) (*n* = 524) at the time of enrolment in the DCMP were excluded. People with an estimated glomerular filtration rate (eGFR) <30 mL/min/1.73 m^2^ (*n* = 72) were also excluded due to its undue effect on HbA1c levels and accuracy of the glycemic status assessment [15]. Ultimately, 3044 individuals were deemed eligible and their data was used for analysis. Based on the propensity score matching procedure as described in the *Statistical analysis* subsection, 757 smokers were further matched with 757 non-smokers in a 1:1 ratio (Figure 1).

### Outcome variable: glycemic control

Glycemic control was assessed using HbA1c values, which were regarded as a continuous variable for analysis. HbA1c levels were measured at enrolment in the DCMP (baseline values) and at 3, 6, 9, and 12 months thereafter by ion-exchange high-performance liquid chromatography using the VARIANTTM II Turbo system.

### Major exposure variable: smoking status

A well-trained certified diabetes educator conducted face-to-face interviews using a computer-assisted standard form to assess and record each person's smoking status at DCMP enrolment and thereafter once a year. All participants were categorised as either smokers or non-smokers based on smoking status at baseline. Smokers were defined as those who had been smoking until enrolment, including social smokers and daily smokers. Non-smokers included those who had never smoked and those who had quit smoking. Considering that availability of baseline smoking status does not represent the continuation of smoking throughout the entire year, data collected at the endpoint were incorporated into a sensitivity analysis, in which participants were divided into four groups: persistent smoking (+/+), persistent non-smoking (–/–), quit smoking (+/–), and irresolute smoking (–/+) (the signs indicated the smoking status at baseline/endpoint) (Figure 1). Based on consumption of cigarettes per day (CPD), smokers were subdivided into heavy smokers (>20 CPD) and light smokers (≤20 CPD).

### Other control variables

Some variables retrieved from the DCMP registry data set were considered control variables, including age at onset of diabetes, education level, family history of diabetes, alcohol consumption, leisure-time physical activity, self-care variables, and physical examination. These data were entered at DCMP enrolment by a certified diabetes educator using a standard assessment form in the electronic medical record system.

Alcohol consumption was defined as more than once weekly within the preceding year. Leisure-time physical activity was classified as regular (≥30 min/day, ≥3 days/week), occasional (less rigorous than regular exercise), or no exercise. A four-point Likert scale was used by a certified diabetes educator to assess the following fours variables of self-care. Knowledge regarding glycemic control was defined as an understanding of the need for and the ways to control blood glucose. Willingness towards DSM was defined as the motivation to learn self-management techniques. Medication adherence was defined as taking the medication regularly at the dose recommended by the physician during the past week. Performing self-monitoring of blood glucose (SMBG) was defined as self-assessment of blood glucose levels using a glucometer more than once per week. Data were merged into simple dichotomies (i.e. top-two-box vs. bottom-two-box) and categorised as either adequate (yes) or inadequate (no) for analysis.

Physical examination included measurement of blood pressure (BP), height, and body weight. Systolic and diastolic BP were measured in a seated position after a 10-minute rest. Body mass index (BMI) was calculated as body weight (kg)/height (m)^2^. Baseline laboratory data included total cholesterol, high-density lipoprotein cholesterol (HDL-C), triglycerides, low-density lipoprotein cholesterol (LDL-C), creatinine, and glutamic pyruvic transaminase levels measured using a UniCel DxC 800 Synchron Clinical System (Beckman Coulter, Brea, CA, USA). eGFR was calculated using the equation recommended by the National Kidney Foundation [16]. Data on 19 major non-psychiatric comorbidities described in the Charlson comorbidity index for the year preceding enrolment were collected from the CCH research database [17]. Major comorbidities, including congestive heart failure, coronary artery disease, and cerebrovascular accidents, were analysed as independent variables. Individual anti-diabetic medication use during the 12-month observation period was divided into three categories: none (no medication use) or oral anti-diabetic drugs (OAD) alone, OAD plus insulin, and insulin alone. Data on all anti-diabetic medications used for ≥1 month were collected and used for analysis.

### Statistical analyses

Data are expressed as participant numbers with percentages or means with standard deviations (SD) for categorical and continuous variables, respectively. Differences between smokers and non-smokers were assessed using the Chi-Square test for categorical variables and the Student's *t*-test for continuous variables. The propensity score was calculated using non-parsimonious multivariable logistic regression and included all variables listed in Table 1. Based on the propensity score, smokers were matched with non-smokers in a 1:1 matching ratio using the nearest-neighbor algorithm with a calliper of 0.1 SD to derive matched pairs.

**Table 1.** Basic characteristics of participants before and after propensity score matching.

	Before propensity score matching			After propensity score matching		
Variables	Smoker (*n* = 892)	Non-smoker (*n* = 2152)	*p*-value	Smoker (*n* = 757)	Non-smoker (*n* = 757)	*p*-value
Number of cigarettes per day	20 (11.9)	--	--	19.2 (11.6)	--	--
≤20 per day	670 (75.1%)	--	--	583 (77.0%)	--	--
>20 per day	222 (24.9%)	--	--	174 (23.0%)	--	--
Age at onset (years)	51.7 (10.9)	56.0 (12.2)	<.001	52.2 (10.9)	52.2 (11.6)	.99
Level of education: No	29( 3.3%)	104 (4.8%)		27 (3.6%)	26 (3.4%)	
Primary school or under	262 (29.4%)	666 (31.0%)	<.001	216 (28.5%)	222 (29.3%)	.96
High school	459 (51.5%)	878 (40.8%)		381 (50.3%)	383 (50.6%)	
University or above	142 (15.9%)	504 (23.4%)		133 (17.6%)	126 (16.6%)	
Family history of DM: Yes	438 (49.1%)	932 (43.3%)	.003	368 (48.6%)	386 (51.0%)	.36
Alcohol drinking	180 (20.2%)	172 (8.0%)	<.001	122 (16.1%)	119 (15.7%)	.83
Physical activity: No exercise	544 (64.6%)	1005 (49.4%)	<.001	467 (61.7%)	462 (61.0%)	.94
Occasional exercise	285 (33.9%)	987 (48.5%)		277 (36.6%)	283 (37.4%)	
Regular exercise	13 (1.5%)	44 (2.2%)		13 (1.7%)	12 (1.6%)	
Knowledge regarding GC: Good	495 (59.9%)	1339 (66.9%)	<.001	465 (61.4%)	463 (61.2%)	.92
Willingness towards DSM: Yes	674 (81.6%)	1699 (84.8%)	.034	624 (82.4%)	635 (83.9%)	.45
Medication adherence: Good	792 (94.4%)	1933 (95.9%)	.073	717 (94.7%)	715 (94.5%)	.82
SMBG: Yes	209 (23.4%)	610 (28.4%)	.005	186 (24.6%)	166 (21.9%)	.22
Clinical variables at baseline						
HbA1c (%)	9.5 (2.8)	9.0 (2.7)	<.001	9.4 (2.7)	9.5 (2.8)	.55
BMI (kg/m^2^)	26.2 (4.2)	26.3 (4.0)	.55	26.2 (4.2)	26.1 (3.9)	.62
SBP (mmHg)	128.2 (17.4)	130.7 (17.0)	<.001	128.4 (17.3)	128.9 (16.2)	.57
DBP (mmHg)	79.4 (11.3)	80.2 (11.2)	.085	79.1 (11.1)	79.5 (10.6)	.54
Total cholesterol (mg/dL)	187.6 (49.0)	178.0 (39.4)	<.001	183.4 (41.3)	182.9 (40.8)	.81
Triglycerides (mg/dL)	207.4 (242.6)	147.9 (128.4)	<.001	181.6 (168.7)	179.8 (180.2)	.84
HDL-C (mg/dL)	41.8 (11.0)	44.5 (10.9)	<.001	42.2 (11.1)	42.3 (9.9)	.86
LDL-C (mg/dL)	109.8 (35.1)	106.1 (32.4)	.008	109.27 (34.1)	108.7 (33.6)	.73
eGFR (mL/min/1.73m^2^)	90.0 (27.9)	85.4 (33.0)	<.001	90 .0 (28.2)	87.9 (26.2)	.14
Anti-hypertensive agents	460 (51.6%)	1254 (58.3%)	.001	393 (51.9%)	411 (54.3%)	.35
Anti-diabetic agents			.29			.85
None	18 (2.0%)	76 (3.53%)		15 (2.0%)	20 (2.6%)	
OAD alone	727 (81.5%)	1746 (81.1%)		616 (81.4%)	612 (80.9%)	
OAD with Insulin	126 (14.1%)	266 (12.4%)		110 (14.5%)	112 (14.8%)	
Insulin alone	21 (2.4%)	64 (3.0%)		16 (2.1%)	13 (1.7%)	
Comorbidity: CCI	1.7 (1.1)	1.9 (1.4)	<.001	1.8 (1.1)	1.8 (1.2)	.65
Congestive heart failure	82 (9.2%)	266 (12.4%)	.012	70 (9.3%)	66 (8.7%)	.72
Coronary artery disease	49 (5.5%)	180 (8.4%)	.006	39 (5.2%)	40 (5.3%)	.91
Cerebrovascular accident	42 (4.7%)	155 (7.2%)	.011	43 (5.7%)	45 (5.9%)	.83
Propensity score	0.38 (0.18)	0.26 (0.14)	<.001	0.35 (0.15)	0.35 (0.15)	.98

Note. Results are expressed as mean (SD) or n (%). Abbreviations: SD: standard deviation; DM: diabetes mellitus; SMBG: self-monitoring of blood glucose; GC: glycemic control; DSM: diabetes self-management; HbA1c: haemoglobin A1c; BMI: body mass index; SBP: systolic blood pressure; DBP: diastolic blood pressure; HDL-C: high-density lipoprotein cholesterol; LDL-C: low-density lipoprotein cholesterol; eGFR: estimated glomerular filtration rate; OAD: oral anti-diabetic drug; CCI: Charlson comorbidity index.

To deal with repeated measurements of HbA1c, generalised estimating equations (GEE) analysis with backward elimination for selecting control variables, was used to estimate the effect of smoking on glycemic control at each time point. Time was taken as a categorical variable with five time points. The reduction of HbA1c from baseline to each time point were compared between smokers and non-smokers in term of the interaction of smoking status and time. The difference in HbA1c reduction between smokers and non-smokers was estimated by the regression coefficient (β) at each time point.

Sensitivity analysis was further conducted to assess the interactions among the four smoking groups categorised according to the smoking status at baseline and endpoint. Subgroup analysis to assess the effect of smoking on HbA1c reduction at each time point was performed in various participant subgroups using GEE, which used repeated HbA1c measurement as the dependent variable and the three-way interaction among smoking status, time, and subgroup covariates as independent variables. All statistical analyses were performed in 2020 using IBM SPSS Statistics version 22.0 (IBM Corp., Armonk, NY, USA), and a two-tailed *p*-value < .05 was considered statistically significant.

## Results

### Characteristics of participants

The study cohort comprised 3044 eligible men with newly diagnosed T2DM who were categorised as either smokers (*n* = 892; 29.3%) or non-smokers (*n* = 2152; 70.7%). Compared to the non-smokers group, the smokers group was younger (51.7 [SD, 10.9] vs 56.0 [SD, 12.2] years, *p* < .001), had a lower education level, had more individuals with a family history of diabetes, had a greater number of alcohol drinkers, had lesser knowledge on glycemic control and willingness towards DSM, had fewer SMBG users and was less physically active at leisure-time (Table 1). No significant differences were found in medication adherence and BMI between the two groups.

Although smokers had higher baseline HbA1c levels and unhealthy blood lipid levels (lower HDL-C, higher total cholesterol, triglycerides and LDL-C levels), they had lower systolic BP, and fewer participants needed anti-hypertensive agents. Additionally, smokers had a lower Charlson comorbidity index, including lower percentages of pre-existing diagnoses of congestive heart failure, coronary artery disease, and cerebrovascular accident than non-smokers. During the 12-month observation period, the use of anti-diabetic medications was not different between the two groups.

As there were significant differences in many characteristics between smokers and non-smokers, a propensity score matched analysis (1:1 match) was carried out wherein 757 participants from the smoker cohort (84.9%) were matched to an equal number of participants in the non-smoker cohort to form the study population (Figure 1). After matching, the characteristics of participants in both cohorts were similar (Table 1).

### Association between smoking status and HbA1c reduction

**Figure 2.**
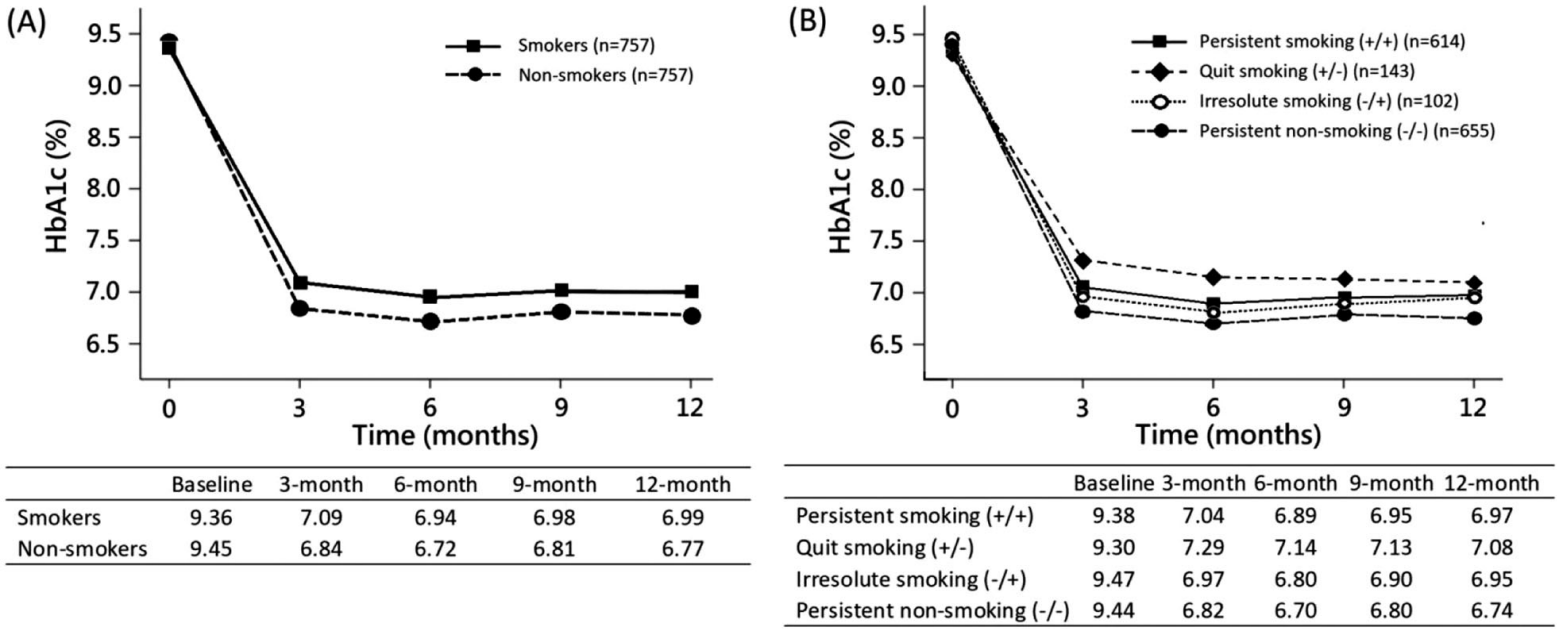
Model-based mean HbA1c levels were estimated by generalised estimating equations in propensity score matched cohorts. (A) Smokers. versus non-smokers. *P*-values for the interaction terms of smoking-by-time were 0.02, 0.04, 0.09 and 0.04 at 3, 6, 9 and 12 months respectively. (B) Four groups according to smoking status at baseline and end-point. Abbreviations: HbA1c, haemoglobin A1c.

Although the matched cohorts showed a reduction in mean HbA1c levels during the observation period, smokers had significantly higher HbA1c levels than non-smokers at 3, 6, 9, and 12 months (Figure 2A, *p*-values for the interaction terms of smoking-by-time were 0.02, 0.04, 0.09 and 0.04 at each time point). Table 2 (model 1) shows the differences in HbA1c reduction between smokers and non-smokers at each time point from baseline, which  were expressed as β of smoking status-by-time interaction in GEE. For example, at 12-months after baseline, non-smokers and smokers had an HbA1c reduction of 2.68% and 2.38%, respectively. The difference in HbA1c reduction between smokers and non-smokers was 0.30%. As a result, smokers had a smaller HbA1c reduction than non-smokers, with the maximum difference of 0.33% (95% CI, 0.05–0.62%) observed at 3 months and the minimum difference of 0.25% (95% CI, −0.04–0.54%) observed at 9 months.

**Table 2.** The association between smoking status and HbA1c reduction was estimated by generalised estimating equations after propensity score matching.

**Model 1:** dichotomous smoking status			**Model 2:** four groups of smoking status		
Variables	*β* (95% CI)	*p*-value	Variables	*β* (95% CI)	*p*-value
**Group:**			**Group:**		
Non-smokers	0		Smoking (−/−)	0	
Smokers	−0.11 (−0.37, 0.16)	.44	Smoking (−/+)	−0.10 (−0.68, 0.47)	.72
			Smoking (+/−)	−0.15 (−0.60, 0.31)	.52
			Smoking (+/+)	−0.11 (−0.40, 0.18)	.45
**Time:** Baseline	0		**Time:** Baseline	0	
3 months	−2.60 (−2.81, −2.40)	<.001	3 months	−2.62 (−2.85, −2.40)	<.001
6 months	−2.73 (−2.94, −2.52)	<.001	6 months	−2.74 (−2.96, −2.51)	<.001
9 months	−2.63 (−2.84, −2.42)	<.001	9 months	−2.64 (−2.87, −2.42)	<.001
12 months	−2.68 (−2.88, −2.47)	<.001	12 months	−2.70 (−2.92, −2.47)	<.001
**Interaction of smoking group and time**			**Interaction of smoking group and time**		
Baseline	0		Smoking (−/+): Baseline 0		
3 months	0.33 (0.05, 0.62)	.02	3 months	0.12 (−0.46, 0.69)	.68
6 months	0.31 (0.02, 0.59)	.04	6 months	0.07 (−0.54, 0.69)	.81
9 months	0.25 (−0.04, 0.54)	.09	9 months	0.07 (−0.57, 0.71)	.84
12 months	0.30 (0.01, 0.59)	.04	12 months	0.18 (−0.44, 0.79)	.58
			Smoking (+/−): Baseline 0		
			3 months	0.62 (0.12, 1.12)	.02
			6 months	0.58 (0.06, 1.10)	.03
			9 months	0.47 (−0.04, 0.99)	.07
			12 months	0.48 (−0.02, 0.98)	.06
			Smoking (+/+): Baseline 0		
			3 months	0.29 (−0.03, 0.60)	.08
			6 months	0.25 (−0.06, 0.57)	.12
			9 months	0.21 (−0.10, 0.52)	.19
			12 months	0.29 (−0.02, 0.60)	.07

Note. The difference in HbA1c reduction between smokers and non-smokers was estimated by the regression coefficient (β) and 95% confidence interval (95% CI) of smoking status-by-time interaction at each time point. The models were established using the backward elimination method to select control variables, including a family history of diabetes, alcohol drinking, knowledge regarding glycemic control, medication adherence, anti-hypertensive agents, blood pressure, total cholesterol, estimated glomerular filtration rate, and body weight change.

Abbreviation: HbA1c: haemoglobin A1c.

The model-based mean HbA1c levels among the four smoking groups categorised according to the smoking status at baseline and endpoint are shown in Figure 2B. Both the persistent non-smoking (−/−) and the irresolute (−/+) groups (which were non-smokers at baseline) had a better HbA1c level than the persistent (+/+) and quit (+/−) smoking groups (which were smokers at baseline) at each time point after enrolment. It is noted that the quit (+/−) smoking group had the worst HbA1c level among them, with a maximum difference of 0.62% (95% CI, 0.12–1.12%) seen at 3 months when compared with persistent non-smoking (−/−) group (Table 2, model 2).

### Dose-response relationship

To analyse any potential dose-response relationship, smokers were subdivided into heavy smokers (>20 CPD) or light smokers (≤20 CPD). Heavy smokers displayed a greater difference in HbA1c reduction (estimated by β of smoking status-by-time interaction) than light smokers, compared to non-smokers, at 6, 9, and 12 months. However, heavy smokers showed a significant difference only at 12 months (0.40% [95% CI, 0.01–0.79%]), while the differences at 3, 6, and 9 months were not significant (Supplementary Table S1 and Figure S1). When taking the amount of smoking (with the three levels) as a continuous variable, the interactions between the smoking amount and each follow-up time-point showed a potential dose-response trend overall. The *p*-values were 0.02, 0.04, 0.07, and 0.04 at 3, 6, 9, and 12 months after enrolment, respectively (results not shown in tables).

### Subgroup analyses

Subgroup analysis was conducted based on the following variables, namely, age at onset of diabetes (<50, ≥50 years), BMI (<25, ≥25 kg/m^2^), eGFR (<60, ≥60 mL/min/1.73 m^2^), family history of diabetes (with, without), SBP (<140, ≥140 mmHg), total cholesterol (<200, ≥200 mg/dL), triglycerides (<150, ≥150 mg/dL), insulin use (with, without), and baseline HbA1c (<7, ≥7%). Only the lower BMI subgroup had significantly greater differences in HbA1c reduction than the higher BMI subgroup at all four time points tested (Table 3).

**Table 3.** Subgroup analysis of the difference in mean HbA1c reduction between smokers and non-smokers at each time point.

Subgroup	*n*	MD at 3-month (95% CI)	*P* for interaction	MD at 6-month (95% CI)	*P* for interaction	MD at 9-month (95% CI)	*P* for interaction	MD at 12-month (95% CI)	*P* for interaction
Age									
<50 years	697	0.39 (0.02, 0.76)	.52	0.32 (−0.04, 0.68 )	.59	0.33 (0.00, 0.67)	.83	0.44 (0.16, 0.71)	.31
≥50 years	817	0.23 (−0.10, 0.55)		0.19 (−0.13, 0.51)		0.28 (−0.02, 0.58)		0.24 (−0.01, 0.49)	
BMI									
<25 kg/m2	659	0.74 (0.35, 1.14)	.002	0.68 (0.30, 1.06)	.002	0.73 (0.37, 1.08)	.001	0.72 (0.43, 1.01)	<.001
≥25 kg/m2	855	−0.04 (−0.35, 0.27)		−0.08 (−0.38, 0.22)		−0.02 (−0.30, 0.26)		0.03 (−0.21, 0.27)	
eGFR									
≥60 mL/min/1.73m^2^	1369	0.32 (0.07, 0.58)	.54	0.28 (0.03, 0.52)	.52	0.33 (0.09, 0.56)	.56	0.38 (0.18, 0.57)	.13
<60 mL/min/1.73m^2^	145	0.06 (−0.80, 0.92)		0.01 (−0.83, 0.85)		0.10 (−0.70, 0.90)		−0.11 (−0.79, 0.56)	
Family history of DM:									
No	760	0.33 (−0.03, 0.69)	.78	0.26 (−0.08, 0.61)	.90	0.34 (0.02, 0.67)	.72	0.32 (0.05, 0.59)	.92
Yes	754	0.27 (−0.07, 0.60)		0.23 (−0.09, 0.56)		0.26 (−0.05, 0.57)		0.34 (0.08, 0.60)	
SBP									
<140 mmHg	1187	0.28 (0.00, 0.56)	.81	0.28 (0.01, 0.56)	.57	0.30 (0.05, 0.55)	.98	0.28 (0.07, 0.49)	.34
≥140 mmHg	327	0.35 (−0.16, 0.87)		0.11 (−0.39, 0.62		0.31 (−0.16, 0.78)		0.50 (0.11, 0.89)	
T-chol									
<200 mg/dL	1040	0.41 (0.12, 0.70)	.23	0.32 (0.04, 0.61)	.44	0.46 (0.19, 0.73)	.05	0.45 (0.23, 0.68)	.06
≥200 mg/dL	474	0.08 (−0.37, 0.53)		0.12 (−0.32, 0.55)		−0.01 (−0.42, 0.39)		0.08 (−0.26, 0.41)	
Triglycerides									
<150 mg/dL	830	0.44 (0.11, 0.77)	.19	0.41 (0.09, 0.74)	.12	0.46 (0.15, 0.76)	.13	0.50 (0.24, 0.76)	.04
≥150 mg/dL	684	0.11 (−0.25, 0.47)		0.03 (−0.31, 0.38)		0.10 (−0.22, 0.43)		0.11 (−0.15, 0.38)	
Non-insulin users	1263	0.32 (0.08, 0.57)	.72	0.29 (0.05, 0.53)	.47	0.31 (0.09, 0.54)	.91	0.35 (0.16, 0.54)	.64
Insulin users	251	0.20 (−0.57, 0.98)		0.06 (−0.70, 0.81)		0.28 (−0.42, 0.97)		0.24 (−0.34, 0.81)	
HbA1c (baseline)									
<7%	355	0.10 (−0.10, 0.31)	.14	0.00 (−0.19, 0.18)	.07	0.08 (−0.09, 0.24)	.07	0.10 (−0.02, 0.23)	.03
≥7%	1159	0.49 (0.22, 0.76)		0.45 (0.19, 0.72)		0.50 (0.25, 0.75)		0.52 (0.31, 0.72)	
Overall patients	1514	0.30 (0.05, 0.55)		0.25(0.01, 0.49)		0.31 (0.08, 0.53)		0.33 (0.15, 0.52)	

Abbreviations: MD: mean difference in HbA1c reduction between smokers and non-smokers; CI: confidence interval; OAD: oral anti-diabetes drug; DM: diabetes mellitus; HbA1c: haemoglobin A1c; BMI: body mass index; SBP: systolic blood pressure; eGFR: estimated glomerular filtration rate; T-Chol: total cholesterol.

## Discussion

We found that among men with newly diagnosed T2DM in a real-world setting, smokers had poorer glycemic control than non-smokers during a 12-month observational period. The difference in HbA1c reduction between smokers and non-smokers was about 0.25% (95% CI, −0.04–0.54%) to 0.33% (95% CI, 0.05–0.62%) and this was even more prominent in smokers with BMI <25 kg/m^2^, 0.68% (95% CI, 0.30–1.06%) to 0.74% (95% CI, 0.35–1.14%) compared to those with BMI ≥25 kg/m^2^, suggesting a stronger detrimental association between smoking and glycemic control in these participants. Further, it shows a potential dose-response trend of the amount of smoking in the reduction of HbA1c, although some of the values did not achieve the significance level.

Many studies have demonstrated an association between smoking and incidence of diabetes (or HbA1c elevation) in the general population without diabetes, while other studies have evaluated people with diabetes to explore the association between smoking and glycemic control, but the latter are mostly cross-sectional studies. Data from the Swedish National Diabetes Registry for 1996–2001 showed that smokers had higher mean HbA1c (unadjusted) levels than non-smokers (6.65% vs 6.44%, *p* < .001) [9]. Similarly, the Fukuoka Diabetes Registry showed that, compared to non-smokers, Japanese male smokers with T2DM had a mean HbA1c (age-adjusted) increase of 0.20% (95% CI, 0.08–0.31%), which also showed a dose-response relationship [10]. A study of 10,551 men with diabetes in China found that smoking was associated with an increased risk (OR: 1.49 [95% CI, 1.35–1.66]) of poor glycemic control (defined as HbA1c ≥7.0%) [12], and that male smokers had higher mean HbA1c levels (unadjusted) than never-smokers (7.82% vs 7.46%, *p* < .001). Another study in China showed that male heavy smokers with T2DM on medical treatment experienced a mean HbA1c increase of 0.38% (95% CI, 0.23–0.53%) compared to non-smokers and that this result exhibited a dose-response relationship [11]. In contrast to these studies, we collected cohort data such that longitudinal analyses could be performed, and our results broadly concur with those reported in the aforementioned studies.

Additionally, notable differences exist between people with diabetes and the general population. For example, when compared to the result of a meta-analysis using data from people without known diabetes that HbA1c was 0.10% (95% CI, 0.08–0.12%) higher in current smokers compared with never-smokers [18], our results suggest a stronger detrimental association between smoking and glycemic control in people with diabetes than the general population.

Several studies demonstrated that T2DM in East Asians is characterised primarily by β cell dysfunction with less adiposity than that in Caucasians. Asians who adopted western dietary habits showed higher rates of diabetes [19,20]. However, studies in either general populations or diabetic patients showed that the detrimental association between smoking and glycemic control is consistent in Asian and Caucasian populations without interethnic differences.

HbA1c is an important indicator of long-term glycemic control. However, Soulimane *et al.* have speculated that techniques used to quantitate HbA1c might be affected by metabolites of tobacco through red blood cells [18], which can affect the interpretation of cross-sectional study results. Thus, the longitudinal nature of our study has potentially overcome this problem while investigating differences in HbA1c reduction between smokers and non-smokers.

Several plausible pathophysiological mechanisms can explain this detrimental effect of smoking on glycemic control, and accumulating scientific evidence has revealed the molecular mechanisms underlying the development of altered glucose homeostasis in smokers [7,8]. Smoking is associated with increased insulin resistance and the possible pathways include visceral fat accumulation [21,22], increased cortisol and thyroid hormone levels [23], increased sympathetic activity [24], and increased systemic inflammation [25]. Studies have also suggested that nicotine exposure could induce a reduction in insulin release and loss of pancreatic beta cell mass, apart from negatively affecting insulin action [7,8].

Some factors may indirectly contribute to this unfavourable glycemic effect. Some studies have found that overlapping unhealthy behaviours in smokers, such as low physical activity, alcohol consumption, and poor diet may result in visceral fat accumulation, which consequently increases their risk of developing T2DM [21,26]. Our study has similar findings, in that, compared to non-smokers, smokers in our cohort were engaged in lesser physical activity, consumed more alcohol, were less motivated for SMBG, and had lower levels of education, lesser knowledge regarding glycemic control, and reduced willingness towards self-management. Crucially, the unfavourable association between smoking and glycemic control persisted even after these confounding factors were essentially adjusted by propensity score matching, suggesting an independent association between smoking and glycemic control.

Subgroup analysis demonstrated that the detrimental association between smoking and glycemic control was even stronger in male smokers with BMI <25 kg/m^2^ than in those with BMI ≥ 25 kg/m^2^. This observation is important as real-world data regarding the association between smoking and glycemic control among people with diabetes stratified by grades of BMI is scarce. Available data on interaction between smoking and BMI on diabetes risk also showed inconsistent results, even though BMI has been recognised as an independent factor associated with diabetes risk in the general population. A meta-analysis of 25 prospective cohort studies has reported that the relative risk of diabetes incidence in smokers with BMI ≥25 was 1.57 (95% CI, 1.35–1.82), while it was 1.34 (95% CI, 1.13–1.58) in those with BMI <25 kg/m^2^, compared to non-smokers with the same BMI grades [4]. Likewise, a study in China has described a strong association between the amount of smoking and the risk of diabetes in people with higher BMI [27]. In contrast, as reported in Rimm *et al.* [28], males with BMI <27.8 kg/m^2^ showed a higher relative risk of diabetes incidence (smokers vs. non-smokers) compared to males with BMI ≥27.8 kg/m^2^. A study in middle-aged Japanese men using a cut-off value of BMI of 24.2 kg/m^2^ also showed a similar finding [29]. A large-scale European study also showed that the association between smoking status and incident diabetes tended to be slightly stronger in men and women without adiposity [30]. Consistent with the results from the latter three studies, we observed a stronger detrimental association between smoking and glycemic control among T2DM men with a lower BMI. Ma *et al.* reported that East Asians had a higher rate of visceral fat than Caucasians at any given BMI. East Asian patients developed T2DM at lower ranges of BMI compared with Caucasians [19]. Therefore, the cut-off point of BMI modifying the association between smoking and glycemic control may have differed from ethnicity.

The strength of our study is its use of longitudinal data to provide evidence of a temporal relationship between smoking status and glycemic control, thereby reducing the possibility of reverse causality, which is a drawback of cross-sectional studies. Consequently, our results represent a more reliable ascertainment of the detrimental association between smoking and glycemic control in men with newly diagnosed T2DM. Furthermore, the use of propensity score matching reduced the possibility of selection bias and addressed the effects of confounders.

Meanwhile, this study has a few limitations. First, selection bias might occur because health behaviours and characteristics between smokers and non-smokers could be different. In the study, propensity score matching has been used to reduce such a potential bias. Second, smokers might have a lower economic status and thus not be able to afford newer or additional medications. This could affect the findings. However, the National Health Insurance in Taiwan covers almost 100% of the population and provides easy access to medical services. Therefore, the treatment or change in medication during the follow-up between smokers and non-smokers was less affected by socioeconomic status. Third, our study included only men with T2DM owing to a very low prevalence of female smokers, which is attributable to the country's cultural background. Therefore, the generalisability of our findings to the whole population should be with caution. Fourth, as a retrospective study, the causal interpretation of this study was limited. Moreover, although propensity score matching was used to improve the comparability of participant characteristics and to minimise selection bias, other unmeasured factors such as dietary habits might affect the selection of controls.

Finally, in our main analysis, participants’ smoking status was classified into 2 categories according to their responses at baseline. Such a measure might not be representative of that throughout the whole year. Cessation of smoking after diabetes had been diagnosed could occur. Taking smoking status at the endpoint into account could somewhat reduce information bias or misclassification of smoking status. As shown, the general results in the sensitivity analysis were consistent with those using smoking data at baseline only. Notably, the quit (+/−) smoking group had an even worse HbA1c level than the persistent smoking (+/+) group. Our observation was in line with previous studies that smoking cessation seems to worsen glycemic control in ex-smokers with diabetes, although the negative effect may decrease with time [31].

## Conclusions

This retrospective cohort study suggests that smoking is independently associated with unfavourable glycemic control among men with newly diagnosed T2DM and that a stronger detrimental association between smoking and glycemic control was observed in men with BMI <25 kg/m^2^ compared to those with BMI ≥25 kg/m^2^. These findings could improve the understanding of smoking in diabetes and facilitate better management of smokers with newly diagnosed diabetes.

## Supplementary Material

Supplemental Material

## Data Availability

The dataset used in the study is not available. Data are confidential according to Personal Information Protection Act implemented by the Taiwanese Government in 2012. Further information on data acquisition is available from the first author upon reasonable request.
