# Supplementary material for: Association between smoking and glycemic control in men with newly diagnosed type 2 diabetes: a retrospective matched cohort study
Source: Ann Med. 2022 May 16;54(1):1385–94. doi: 10.1080/07853890.2022.2075559 (PMC9126565; doi:10.1080/07853890.2022.2075559)
Supplement: Supplemental Material [file IANN_A_2075559_SM0624.zip › Supplemental files/supplementary Figure S1_0324.pdf]

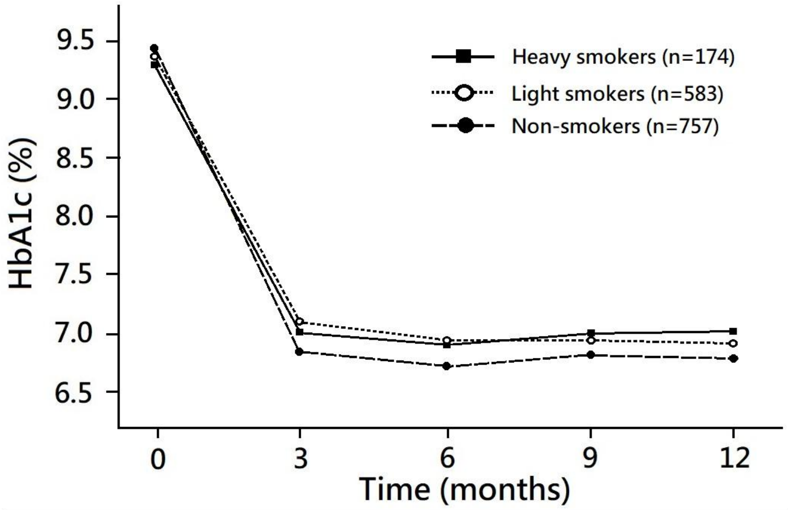

	Baseline	3-month	6-month	9-month	12-month
Heavy smokers	9.30	7.02	6.89	7.00	7.02
Light smokers	9.37	7.11	6.94	6.94	6.93
Non-smokers	9.45	6.84	6.72	6.81	6.77

Supplementary Figure S1. Model-based mean HbA1c levels estimated by generalized estimating equations in propensity score matched cohorts. Smokers were subdivided as heavy smokers (>20 CPD) or light smokers (≤20 CPD).

Abbreviations: HbA1c, hemoglobin A1c; CPD, cigarettes per day.
