# Supplementary material for: Association between smoking and glycemic control in men with newly diagnosed type 2 diabetes: a retrospective matched cohort study
Source: Ann Med. 2022 May 16;54(1):1385–94. doi: 10.1080/07853890.2022.2075559 (PMC9126565; doi:10.1080/07853890.2022.2075559)
Supplement: Supplemental Material [file IANN_A_2075559_SM0624.zip › Supplemental files/supplementary Table S1_0324.docx]

**Supplementary Table S1.** Dose-response relationship between smoking status and HbA1c reduction estimated by generalized estimating equations after propensity score matching

| **Variables** | β (95% CI) | p-value |
| --- | --- | --- |
| **Group:** Non-smokers | 0 |  |
| CPD ≤20 | -0.08 (-0.38 to 0.22) | 0.62 |
| CPD >20 | -0.15 (-0.50 to 0.21) | 0.42 |
| **Time:** Baseline | 0 |  |
| 3 months | -2.60 (-2.81 to -2.40) | <0.001 |
| 6 months | -2.73 (-2.94 to -2.52) | <0.001 |
| 9 months | -2.63 (-2.84 to -2.42) | <0.001 |
| 12 months | -2.68 (-2.88 to -2.47) | <0.001 |
| **Interaction of smoking group and time** | | |
| CPD ≤20: Baseline | 0 |  |
| 3 months | 0.34 (0.02–0.66) | 0.04 |
| 6 months | 0.30 (-0.03–0.62) | 0.07 |
| 9 months | 0.20 (-0.13 to 0.52) | 0.23 |
| 12 months | 0.24 (-0.09–0.56) | 0.15 |
| CPD >20: Baseline | 0 |  |
| 3 months | 0.32 (-0.06–0.71) | 0.10 |
| 6 months | 0.32 (-0.08 to 0.72) | 0.12 |
| 9 months | 0.33 (-0.06 to 0.72) | 0.09 |
| 12 months | 0.40 (0.01 to 0.79) | 0.04 |

Note. The difference in HbA1c reduction between smokers and non-smokers was estimated by the regression coefficient (β) and 95% confidence interval (95% CI) of smoking status-by-time interaction at each time point. The models were established using the backward elimination method to select control variables, including family history of diabetes, alcohol drinking, knowledge regarding glycemic control, medication adherence, anti-hypertensive agents, blood pressure, total cholesterol, estimated glomerular filtration rate, and body weight change.

Abbreviations: HbA1c, hemoglobin A1c; CPD, number of cigarettes per day.
